# Exploring undergraduate midwifery students’ readiness to deliver culturally secure care for pregnant and birthing Aboriginal women

**DOI:** 10.1186/s12909-015-0360-z

**Published:** 2015-04-16

**Authors:** Rosalie D Thackrah, Sandra C Thompson, Angela Durey

**Affiliations:** 1 Western Australian Centre for Rural Health, University of Western Australia, Perth, Western Australia Australia; 2 Faculty of Health Sciences, Curtin University, Perth, Western Australia Australia; 3 School of Dentistry, University of Western Australia, Perth, Western Australia Australia; 4 Centre for Aboriginal Studies, Curtin University, Perth, Western Australia Australia

**Keywords:** Midwifery education, Aboriginal health, Culturally secure practice

## Abstract

**Background:**

Culturally secure health care settings enhance accessibility by Aboriginal Australians and improve their satisfaction with service delivery. A culturally secure health service recognises and responds to the legitimate cultural rights of the recipients of care. Focus is upon the health care system as well as the practice and behaviours of the individuals within it. In an attempt to produce culturally secure practitioners, the inclusion of Aboriginal content in health professional programs at Australian universities is now widespread. Studies of medical students have identified the positive impact of this content on knowledge and attitudes towards Aboriginal people but relatively little is known about the responses of students in other health professional education programs. This study explored undergraduate midwifery students’ knowledge and attitudes towards Aboriginal people, and the impact of Aboriginal content in their program.

**Methods:**

The study surveyed 44 students who were in their first, second and third years of a direct entry, undergraduate midwifery program at a Western Australian (WA) university. The first year students were surveyed before and after completion of a compulsory Aboriginal health unit. Second and third year students who had already completed the unit were surveyed at the end of their academic year.

**Results:**

Pre- and post-unit responses revealed a positive shift in first year students’ knowledge and attitudes towards Aboriginal people and evidence that teaching in the unit was largely responsible for this shift. A comparison of post-unit responses with those from students in subsequent years of their program revealed a significant decline in knowledge about Aboriginal issues, attitudes towards Aboriginal people and the influence of the unit on their views. Despite this, all students indicated a strong interest in more clinical exposure to Aboriginal settings.

**Conclusions:**

The inclusion of a unit on Aboriginal health in an undergraduate midwifery program has been shown to enhance knowledge and shift attitudes towards Aboriginal people in a positive direction. These gains may not be sustained, however, without vertical integration of content and reinforcement throughout the program. Additional midwifery-specific Aboriginal content related to pregnancy and birthing, and recognition of strong student interest in clinical placements in Aboriginal settings provide opportunities for future curriculum development.

## Background

Professional accreditation standards in medicine and the health sciences increasingly require that practitioners be ‘culturally competent’ although there remains uncertainty about how best to measure this attribute, and if acquired, the extent to which it is translated into clinical practice. Debate also surrounds the concept itself with other terms such as cultural respect and cultural security often used interchangeably with cultural competence. However, following the 2011 recommendations from Universities Australia [1], the peak body representing all national universities, the notion of ‘Indigenous^a^ cultural competency’ as a graduate attribute has gained momentum in the higher education sector (see Endnote^a^ for an explanation of terminology). In their report outlining the guiding principles for the development of ‘Indigenous cultural competency’, the authors defined the concept as ‘student and staff knowledge and understanding of Indigenous Australian cultures, histories and contemporary realities and awareness of Indigenous protocols, combined with the proficiency to engage and work effectively in Indigenous contexts congruent to the expectations of Indigenous Australian peoples’ [1].

Developments within universities and regulatory bodies paralleled changes in Australia’s social and political climate where reconciliation with Aboriginal^a^ people, the National Apology to the Stolen Generations and the ‘Closing the Gap’ campaign created heightened awareness of socio-economic and health disparities between Aboriginal and non-Aboriginal Australians [2]. Increased utilisation of health care services and ultimately improved health status are anticipated outcomes of a culturally competent health care workforce, although there is limited evidence linking cultural competence to improved health outcomes and a reduction in health disparities [3-5].

Even prior to Universities Australia’s report, efforts to develop more culturally inclusive curricula were proceeding in medicine, nursing, midwifery and allied health programs, albeit at different rates. In 2004 the Committee of Deans of Australasian Medical Schools (CDAMS) ‘Indigenous Health Curriculum Framework’ [6] identified key principles with respect to core Aboriginal content. These included diversity within and between communities; the validity of Aboriginal views on health and wellbeing; the importance of Aboriginal professionals’ and community members’ knowledge and expertise in curriculum development; and the contribution that a culturally safe medical workforce can make to improving Aboriginal health outcomes [6]. Furthermore, a positive strengths-based model was encouraged together with vertical and horizontal integration of Aboriginal content. The CDAMS framework was subsequently included in accreditation guidelines and a 2012 audit revealed a significant increase in the amount of Aboriginal content in medical schools over an eight year period, despite variability in ‘comprehensiveness and effectiveness’ [7].

Curriculum initiatives and national competencies in nursing, midwifery and the allied health professions mirrored developments in medicine although were implemented later. The Congress of Aboriginal and Torres Strait Islander Nurses (CATSIN) lobbied strongly for the inclusion of compulsory Aboriginal content in nursing curricula. The ‘gettin em n keepin em’ report which confirmed that few nursing schools integrated core content into programs, made numerous recommendations with respect to revising curriculum frameworks in nursing and midwifery [8]. The profession of midwifery was, until quite recently, subsumed under the ‘nursing’ label, however, nursing councils, boards and academic schools now commonly identify midwifery as a profession in its own right. CATSIN too, has recently changed its title to CATSINaM (aM = and Midwifery) reflecting this trend. This is significant as both professions are now required by regulatory bodies to include core Aboriginal content in their programs although their focus differs, and this is not necessarily reflected in the generic Aboriginal units offered. Programs in psychology, physiotherapy and occupational therapy have incorporated Aboriginal content in their curricula for some time but attempts are now being made to streamline offerings following completion of the national ‘Aboriginal and Torres Strait Islander Health Curriculum Framework’ in early 2015 [9].

Evaluation of Aboriginal health curriculum initiatives in medical school programs has been conducted although according to the National Medical Education Review [7] reporting has been inconsistent. Early findings from an on-going evaluation at the University of Western Australia found that ‘. . . significant shifts in self-perceived levels of knowledge, skills and attitudes . . .’ could be achieved from carefully structured and targeted teaching and learning in the area of Aboriginal health [10]. Integration of material presented, involvement of Aboriginal people in the design of the content and teaching, and the experience of staff were identified as key factors in the achievement of learning outcomes [10]. Subsequent studies investigated the role of reflective practice in the assessment process, particularly as it applied to the preparation of case histories with Aboriginal patients. Ewen, Paul and Bloom suggested that an understanding of the context of interactions between Aboriginal patients and health professionals and the consequent impact on health outcomes is a serious omission from many health science programs [4]. Other studies have explored medical student responses to Aboriginal content, their interactions with Aboriginal patients, and the benefits of cultural immersion experiences [11-13].

Limited evaluation of the impact of Aboriginal content in nursing, midwifery and allied health programs has been reported but is expected to increase when the ‘Aboriginal and Torres Strait Islander Health Curriculum Framework’ is implemented [14-18]. Substantial variations exist in the degree of integration of Aboriginal content in programs, the involvement of Aboriginal people in its development and delivery, and the number of hours dedicated to Aboriginal health over the course of a program [1]. Furthermore, little is known about the extent to which student learnings are reinforced throughout programs and transferred into clinical practice upon entry to the workforce. Such knowledge is fundamental to understanding the potential relationship between cultural competency and improved health outcomes for Aboriginal patients.

In this study, a core undergraduate Aboriginal health unit designed and taught by a team of Aboriginal and non-Aboriginal academics, was introduced into an interprofessional common first year in a health science faculty at a Western Australian university. Unit development drew upon an earlier iteration that was introduced into nursing and midwifery programs in 2006 and 2008 respectively. It was well received and recognised with an Australian Learning and Teaching Council Award in 2010.^b^ The new unit, upon which this study is based, was introduced in 2011 and is now undertaken by over 2000 interprofessional first year students. In 2014 this unit also received national recognition. Both units used as their frame of reference the concepts of cultural safety and cultural security (J. Hoffman, personal communication, June 2013). These concepts, with their emphasis on the recipients of care and the importance of Aboriginal cultural values in health service delivery, were reinforced throughout 12 two-hour tutorial sessions across the semester. The new unit utilised specially prepared vodcasts featuring Aboriginal speakers and adhered to a tightly structured format of discussion and presentations. Content included diversity within Aboriginal communities and international comparisons, past policies and practices, social determinants of health, family structures and responsibilities, cultural health beliefs and professional practice issues. For the first year midwifery students, an experienced non-Aboriginal tutor taught the unit; second year students who participated in this study had also been taught by this tutor. Assessment was based on student presentations, online quizzes and submission of a reflective journal.

Given that Aboriginal women have higher fertility and morbidity rates than non-Aboriginal women, their infants have poorer health outcomes, and there is reluctance by some women to use mainstream services [19,20], it is timely to investigate the preparation of midwifery students to provide culturally secure care in clinical practice. This paper presents research that explored midwifery students’ knowledge and attitudes towards Aboriginal people, and the impact of the Aboriginal unit in their program. The findings form part of a larger study which utilised classroom observations and in-depth interviews with students to investigate culturally secure practice in midwifery education and service provision for Aboriginal women. Together, the findings add to the limited research on attitude change and students’ perceptions of their knowledge and readiness to work in Aboriginal contexts.

## Methods

Information was gathered from students using pre-tested questionnaires that included validated items from past medical student and psychology studies [10,13,21] and newly designed questions relevant to midwifery students. (The corresponding author can be contacted for copies of the questionnaires). Permission was granted to include previously validated items in the midwifery student survey. Approval for the study was granted by the Western Australian Aboriginal Health Ethics Committee and the Human Research Ethics Committees at the University of Western Australia and Curtin University.

The lead researcher, with the permission and support of academic midwifery staff, spoke to students about the study, obtained written consent and distributed and collected the questionnaires. A personal approach was utilised reflecting the researcher’s desire to provide a context for the study and address any questions raised, although questionnaires were e-mailed when students were absent from class. The first year students were surveyed before and after completion of a compulsory Aboriginal health unit. Second and third year students who had already completed the unit were surveyed at the end of their academic year. The researcher, a longstanding staff member, had previously taught midwifery students but excluded herself from teaching in the program at the time of the study.

Students responded to a series of statements about perceptions of their knowledge regarding Aboriginal issues; recorded their attitudes towards Aboriginal Australians using an attitude thermometer; identified factors that shaped their attitudes; and indicated their expected involvement with Aboriginal patients in the future. They also responded to 16 statements about Aboriginal health used in previous medical student studies [10]. Survey data were coded and analysed using the Statistical Package for the Social Sciences (SPSS). Due to small numbers, analysis was largely descriptive, although statistical testing (Wilcoxon Signed Rank Test) that compared matched pre- and post-unit responses and post-unit responses with the combined second and third year responses was undertaken. Opened-ended questions were categorised and thematically analysed [22].

## Results

The undergraduate cohort comprised 47 students, 44 of whom completed questionnaires. The three-year undergraduate direct entry program accepted a maximum of only 20 students annually. Four students were exempt from the unit due to recognition of prior learning in Aboriginal health and were not included in the study population. The first year students (n = 16) were surveyed immediately before the commencement of the unit and then upon completion following 12 weeks of instruction. The post-unit questionnaire was completed by 12 of the initial 16 students. The second and third year students (n = 31) completed the unit in the first year of their program. Twenty-eight of the 31 students completed the questionnaire and recalled the impact of the unit retrospectively.

### Undergraduate student demographic characteristics

The undergraduate age distribution reflected the tendency for fewer ‘straight from school’ students to enrol in the direct entry midwifery program compared with nursing and allied health programs. Only 7 students (16%) were aged 17–20 years and 9 (20%) were aged over 40 years. Forty per cent of students (18) had a prior tertiary qualification. Eighty per cent of students were Australian-born, and three acknowledged Aboriginal descent although only one identified as Aboriginal. All overseas-born students were from English-speaking countries, most commonly, England and Ireland. The majority of Australian-born students were raised in large cities and only 4 (10%) spent their childhood in small country towns or remote communities. This urban focus was also reflected in students’ desire to practice in the metropolitan area of Perth or in large regional centres in their first five years following graduation, with only 2 students indicating interest in small, remote settings. The majority identified public hospital services as their preferred employment setting with community-based midwifery the second most favoured option. One student identified Aboriginal-focused practice as a first preference and three as a second preference for employment.

### First year pre- and post-unit survey responses

Despite small numbers, students’ knowledge about issues facing Aboriginal people, Aboriginal history and culture, and Aboriginal health was significantly greater after completion of the unit. Relative to pre-unit responses, no students considered their knowledge ‘less than adequate’ after completion of the unit and those who considered their knowledge ‘more than adequate’ increased. Pre-unit and post-unit self-reported knowledge about Aboriginal issues is presented in Table 1.

**Table 1:** **Pre-unit and post-unit students’ self-reported knowledge about Aboriginal issues**

Knowledge statements	First year pre-unit student responses (A=Adequate)				First year post-unit student responses (A=Adequate)				Change in score from baseline			Wilcoxon signed rank test results
	Less than adequate	A	More than adequate	N	Less than adequate	A	More than adequate	N	Less than adequate	A	More than adequate	
How do you rate your knowledge of issues facing Aboriginal people?	9	5	2	**16**	0	2	10	**12**	-9	3	+8	T = 66
												z = -3.017
												N-ties=11
												**p = .003**
How do you rate your knowledge of Aboriginal history and culture?	9	4	3	**16**	0	4	8	**12**	-9	0	+5	T=55
												z= -2.88
												N-ties=10
												**p = .004**
How do you rate your knowledge of Aboriginal health?	9	6	1	**16**	0	5	7	**12**	-9	-1	+6	T=55
												z = -2.87
												N-ties=10
												**p = .004**

**Key:** T = sum of ranks; z = +/−1.96 or greater to reach statistical significance (p = .05); N = number of participants.

Attitudes towards Aboriginal people were self-reported on an attitude thermometer which provided 10° intervals ranging from 0° (extremely unfavourable) to 100° (extremely favourable), with the mid-point of 50° representing neither favourable nor unfavourable attitudes. Individual pre- and post-unit responses are presented in Table 2.

**Table 2:** **Pre-unit and post-unit students’ self-reported attitudes towards Aboriginal people**

Student ID	1	2	3	4	5	6	7	8	9	10	11	12	13	14	15	16	Mean	SD
**Pre unit**	25°	80°	n/r^*^	30°	95°	50°	50°	60°	70°	100°	80°	50°	50°	75°	70°	60°	63°	21.5°
**Post-unit**	**65**°	**100**°	**50**°	**40**°	**n/r**	**n/r**	**n/r**	**80**°	**100**°	**90**°	**80**°	**90**°	**80**°	**90**°	**n/r**	**70**	**78**°	**18.7**°
**Change from baseline**	+40	+20	-	+10	-	-	-	+20	+30	−10	0	+40	+30	+15	-	+10	+15	−2.8

**Scale: 0**° **=** extremely unfavourable **100**° **=** extremely favourable.

**SD** = Standard deviation; ° = degrees; *n/r = no response/missing.

Students’ attitudes as measured by the attitude thermometer were significantly more positive after completion of the unit (z = −2.61; p = .009). Relative to pre-unit rankings, 9 students rated their attitude towards Aboriginal Australians more highly with only one dropping (from 100 to 90) and one remained unchanged. When students reported factors that shaped their attitudes towards Aboriginal people and understanding of Aboriginal issues, ‘lived experience’, ‘family’ and ‘media’ were identified as the most influential factors in the pre-unit responses. Post-unit responses identified ‘teaching in midwifery course’, closely followed by ‘lived experience’ and ‘family’. These differences did not reach statistical significance. With respect to expected involvement with Aboriginal patients upon graduation, pre- and post-unit responses remained the same with just over 40 per cent of students expecting involvement to be ‘quite often’ or ‘frequent’.

Responses to a series of 16 statements on Aboriginal health provided further evidence of the positive impact of the unit on students. Pre- and post-unit changes were observed with respect to students’ perceptions of their capacity to communicate with Aboriginal women (z = −2.81; p = .005) and to encourage adherence to advice offered (z = −2.48; p = .013). Students were also more likely to agree with the statement ‘The state of Aboriginal health is mainly due to a lack of funding for health services’ following completion of the unit (z = −2.23; p = .025) which suggested acknowledgement of structural factors at play in health status. Responses to other statements did not reach statistical significance however, upon completion of the unit all but two students indicated that the content had changed their views on Aboriginal issues (most expected it would). With only one exception all agreed that an Aboriginal health unit should be compulsory in all health science courses; they would work towards improvements in Aboriginal health as a personal priority; and they had a social responsibility to work towards changes in Aboriginal health. Pre-unit responses indicated that the majority of students held these views before they commenced the Aboriginal health unit so little change was observed.

Upon completion of the unit, students were asked to comment on their receptivity to the content, the extent to which it influenced their perceptions of Aboriginal communities and their health, the adequacy of the amount of teaching in the area, and interest in more clinical exposure to Aboriginal settings. Seventy-five per cent (9 students) indicated that they were ‘receptive’ or ‘very receptive, would like more content in program’, while the remaining three noted that they were ‘resistant at first but this changed over the semester’. Half of the students also reported that teaching in the Aboriginal health unit had ‘considerable influence’ on their perceptions of Aboriginal people while the others reported ‘some or reasonable influence’. This is consistent with the finding from the attitude statements that content in the unit had changed student views on Aboriginal issues. With respect to the adequacy of the amount of teaching in Aboriginal health for their level of training, 84 per cent of students (10) considered it to be ‘adequate’ or ‘more than adequate’ and over 90 per cent (11) expressed interest in more clinical exposure to Aboriginal settings in their training.

### Combined undergraduate post-unit survey responses

Undergraduate responses were combined to determine the overall impact of the Aboriginal health unit on students and to compare responses of students immediately upon completion with those who completed the unit in preceding years. Significant differences were observed between post-unit first year students and the combined group with respect to knowledge about issues facing Aboriginal people. Although the second and third year students had previously completed a unit on Aboriginal health, they were less likely than the first years to rate their knowledge as adequate or more than adequate (z = −2.542; p = 0.011). This pattern was repeated with respect to their knowledge of Aboriginal history, culture and health although did not reach statistical significance. Twenty-five per cent of the second and third year students (7) considered their knowledge about Aboriginal health to be inadequate, compared with no post-unit first year students. A breakdown by year group reveals a gradual drop off in confidence, although the small numbers prevented further analysis.

Self-reported attitudes towards Aboriginal people for the three undergraduate groups produced a mean of 68° based on raw scores. A break down by year group and categories (Table 3) shows that while 83% of students (10/12) who had just completed the Aboriginal unit reported ‘highly favourable’ or ‘favourable’ attitudes, this dropped to 64% for second year students (9/14) and was only 36% for third year students (4/11). Differences also were observed in ‘unfavourable’ and non-committal responses, especially among final year students. Means calculated from raw scores equalled 78° for first year post-unit students, but were lower at 69° for second year students and 55.5° for final year students.

**Table 3:** **Students’ self-reported attitudes towards Aboriginal people by year group**

Attitude	1st year post unit students (N = 12)	2nd year students (N = 15)	3rd year students (N = 13)
Highly favourable 90° – 100°	5	4	1
**Favourable 60**° **- 80**°	**5**	**5**	**3**
Neither favourable or unfavourable 50°	1	2	4
**Unfavourable 20**° **- 40**°	**1**	**3**	**3**
Highly unfavourable 0° - 10°	0	0	0
**TOTAL**	**12**	**14** ^**1**^	**11** ^**2**^

**Note:**^1^One missing case in 2nd years; ^2^Two missing cases in 3rd years; ° = degrees.

Differences in raw scores between first year students and combined second and third year students were statistically significant (z = −1.95, p = .051). When second and third students ranked factors which shaped their attitudes towards Aboriginal people and understanding of Aboriginal issues, they identified ‘lived experience’ as the most influential factor, followed by ‘teaching in midwifery program’. First year post-unit students reversed the order of these factors and were more likely to be influenced by teaching in the Aboriginal health unit. This difference was statistically significant (z = −2.412, p = .016).

Responses to the 16 statements on Aboriginal health did not vary greatly across groups. Almost all students recognised Aboriginal health as a social priority, trust as an essential attribute of culturally secure care, and intimidation as a barrier. When first year post-unit student responses were compared with the combined second and third year groups, only two statements differentiated them statistically: ‘The state of Aboriginal health is mainly due to lack of funding for health services’ (z = −1.95, p = .051) and ‘The information I learned in this unit has changed my views on Aboriginal issues’ (z = −2.465, p = .014) with first year post-unit students more likely to agree with these statements. Undergraduate student responses to Aboriginal-related statements combined for the three year groups are presented in Table 4.

**Table 4:** **Undergraduate midwifery student responses to statements about Aboriginal-related issues (adapted from Paul, Carr and Milroy** [10]**)**

Social priority statements	Undergrad responses % N = 40		
	Agree	Disagree	Neither
The state of Aboriginal health is a social priority.	97.5	2.5	0
Trust is a key for culturally secure health care.	95.0	2.5	2.5
Feeling intimidated is a barrier to culturally secure health care.	95.0	2.5	2.5
**Health service delivery statements**			
The Western medical model suits the health needs of Aboriginal peoples.	7.5	82.5	10.0
The state of Aboriginal health is mainly due to a lack of funding for health services.	22.5	52.5	5.0
Aboriginal people have the same level of access to health services as all other Australians.	20.0	67.5	12.5
Community control in Aboriginal health care services is fundamental to the improvement of health for Aboriginal people.	75.0	7.5	17.5
Aboriginal people should take more individual responsibility for improving their own health.	80.0	2.5	17.5
**Preparedness and ability statements**			
I think it will be difficult to get Aboriginal women to adhere to advice from health professionals.	62.5	10.0	27.5
I think I have the ability to communicate with Aboriginal women by myself.	62.5	10.0	27.5
I need to think beyond the individual when considering Aboriginal health issues.	92.5	0	7.5
The information I learned in the Indigenous Cultures and Health unit changed my views on Aboriginal issues.	65.0	12.5	22.5
It is important that a unit on Indigenous Cultures and Health is compulsory in all health science courses.	90.0	5.0	5.0
**Future commitment statements**			
I will work for improvements in Aboriginal health as a personal priority in my health practice.	70.0	5.0	25.0
I have a social responsibility to work for changes in Aboriginal health.	80.0	5.0	15.0

When all undergraduate students were asked to comment on their responses to Aboriginal health content delivered in the midwifery program, the majority (80%) indicated they were either ‘generally receptive’ (44%) or ‘very receptive, want more content in program’ (36%). Just over half of the students (22) indicated that their perceptions about Aboriginal communities and health had been ‘reasonably or considerably influenced’ by teaching in the area. When first year responses were compared with the combined second and third years, the differences were statistically significant (z = −2.875; p = .004) with teaching having more influence upon perceptions among those who had just completed the unit.

With respect to the adequacy of the amount of teaching received in Aboriginal health for their level of training, 75 per cent of students (30) indicated that it was ‘adequate’ or ‘more than adequate’. Differences were not observed between first years and the combined second and third year group. In addition, 85 per cent of third year students (13) considered they were ‘adequately’ or ‘more than adequately’ prepared to work with Aboriginal women and their babies, a question asked only of this group who were about to graduate. There was a consistent desire among all students for more clinical exposure in Aboriginal settings, with over 80 per cent (32) indicating interest.

Lastly, when students were asked to comment in an open-ended question about topics they would like covered in additional teaching on Aboriginal health the most frequently cited suggestion was for more information on Aboriginal cultural protocols surrounding pregnancy, birth and the post-partum period. Students wanted to learn about midwifery-specific issues including birthing on lands, women’s business, birthing beliefs and practices and grandmothers’ law^c^. Reference also was made to communication skills, which many students considered were undeveloped.

## Discussion

Australian National Competency Standards for the Midwife [23] require that midwifery practice be ‘culturally safe’, a concept developed in New Zealand in the late 1980s. Central to the concept of cultural safety and the associated term cultural security, is recognition of and respect for cultural values in clinical settings. The presence and effectiveness of culturally safe health care is determined by the recipients of care, with unsafe cultural practice viewed as ‘. . . any action which diminishes, demeans, or disempowers the cultural identity and well-being of an individual’ [24,25]. Similarly, the concept of cultural security developed in the Australian context requires that health services ‘. . . not compromise the legitimate cultural rights, values and expectations of Aboriginal people . . .’ [26] and as Houston noted, the crux of cultural security is ‘. . . a shift in emphasis from attitude to behaviour’ [27].

In an effort to prepare culturally safe practitioners, Australian undergraduate midwifery programs are required to include a compulsory unit on Aboriginal health. Competency standards make reference to content including historical background, variations in cultural meanings and responses to health and maternity care, specific health needs of Aboriginal women and recognition and respect for customary law [23]. In this study, students completed an Aboriginal health unit in the second semester of the first year of their program. This unit was the principal source of information on Aboriginal health and cultural issues as there was little integration of content throughout the three year program.

### Enhancing knowledge and shifting attitudes: immediate impact of the Aboriginal unit

Findings from the first year student surveys conducted immediately before and after completion of the Aboriginal unit revealed a positive shift in knowledge and attitudes towards Aboriginal people and evidence that teaching in the unit was largely responsible for this shift. Receptivity to unit content was high, and all students acknowledged its influence on their perceptions about Aboriginal communities and their health. Given that prior to the unit the majority of students had little contact with or knowledge about Aboriginal people and the impact of colonisation on their communities, it is unsurprising that they responded emotionally to the content delivered. Some revealed a deep sense of sadness and shame about our shared history [18] and similar responses to Aboriginal content in health science and medical programs have been reported elsewhere [12,16].

Enhanced knowledge about the causes of disadvantage has been linked with more positive attitudes towards Aboriginal people and their communities [10,12,13,28] and the findings from the pre- and post-unit surveys reinforced this observation. While the small class size was a limitation and the findings require cautious interpretation, individual shifts in knowledge and attitudes did prove statistically significant. On completion of the unit students rated their knowledge of issues facing Aboriginal people, including history, culture and health, more highly than before they commenced the unit and self-reported attitudes towards Aboriginal people were more positive. So what does this tell us? While the survey findings suggest that the Aboriginal unit successfully enhanced students’ knowledge and shifted attitudes towards Aboriginal people, for many it was an unsettling learning experience. Findings reported elsewhere reveal that students grappled with their own pre-existing attitudes, the various factors that shaped them and the responses of their peers to the content [18]. Open-ended questionnaire responses, interviews and classroom observations identified the presence of underlying tensions and unresolved issues, especially related to racism, despite the positive impact of the unit [18].

McDermott has identified the challenges involved in responding to the health consequences of racism in classroom settings, including resistance on the part of students [29]. An evaluation of the impact of integrated Aboriginal health curriculum initiatives on medical students at the University of Western Australia, also found that while changes in self-perceived levels of knowledge, skills and attitudes were possible, the process of building a culturally secure health care workforce remained a challenging and complex endeavour [10]. When interpreting the findings from survey data in this study it is important to recognise this complexity and the discomfort many students encountered during the process of knowledge acquisition and attitude transformation.

### Maximising gains and preparation for practice

Few studies have investigated the longer term impact of Aboriginal content in medical and health science programs although a number have explored student responses to content and perceptions of their abilities to work with Aboriginal patients [10,12,13,16]. Evaluation of the impact of educational interventions on attitudes and behaviours in professional practice settings has been identified as essential to determine whether cultural competence strategies in curricula reduce disparities in health care outcomes [3,4]. Any ‘sustained’ impact of Aboriginal health content is often viewed in this context, that is, translation to clinical practice. However, this small cross-sectional study suggests that even before students enter professional practice, the gains made in knowledge acquisition and attitude change that followed exposure to Aboriginal content may not be sustained as students progress through a program. Not only was a decline in students’ knowledge and attitudes towards Aboriginal people observed across year groups, a statistical difference with respect to the influence of the unit on shaping attitudes toward Aboriginal people was also found, with first year students identifying a stronger impact. This finding, if replicated in a larger, longitudinal study where the same students were surveyed in their first, second and final years, would bring into question the placement of an Aboriginal health unit early in a program, particularly in the absence of integrated content in subsequent years.

It can be speculated that a number of factors contributed to this pattern of findings, although further investigation is required. Certainly many first year students were deeply affected by the content in the Aboriginal health unit, especially the experiences portrayed by Aboriginal people in vodcasts. Students were challenged to question stereotypes, confronted with examples of racism in the health care system and required to reflect on their own attitude formation [18]. The profound nature of the content may explain the immediate impact on students, and it would not be surprising if this impact was diluted over time if content was not reinforced through careful integration in subsequent years of the program. Vertical integration of Aboriginal content in curricula in medical programs has been shown to produce significant improvements in the preparedness of students to work with Aboriginal people [30]. Opportunities for vertical integration would be afforded by the inclusion of additional midwifery-specific content that students suggested, including Aboriginal cultural protocols surrounding pregnancy, birth and the post-partum period.

Finally, the inevitable priority given to other aspects of midwifery practice throughout the program and the process of retrospectivity where students recalled the impact of the Aboriginal unit, may have diluted the importance and retention of content delivered in the first year. It could also be argued that the more exposure students had to pregnant and birthing Aboriginal women in clinical practice, the more they realised how little they really knew. However, this does not account for the significant decline in positive attitudes towards Aboriginal people observed or the fact that the majority of final year students considered they were adequately prepared to work with Aboriginal women.

While there remain unanswered questions that require further investigation in a larger study, it is clear that more attention should be given to maximising the positive gains in students’ knowledge and attitudes observed following exposure to the Aboriginal unit. This might be achieved through better vertical integration of content or the location of unit content in a later year of the program. The provision of more clinical practice opportunities with Aboriginal women, an idea supported by the overwhelming majority of students, seems likely to strengthen preparation to deliver culturally secure care. While further research is required to investigate the transferability of student learnings into professional practice and to determine whether culturally secure practice is associated with better Aboriginal health outcomes, responses to the teaching of such content remain vitally important as part of the process and warrant closer scrutiny. It is in the academy where future health professionals are trained and this setting provides an excellent opportunity to influence the development of a culturally secure workforce.

## Conclusions

Although Aboriginal content in medical and health sciences programs in Australian universities is widespread, the amount of content, its placement in a program and the degree of vertical integration differs widely and may impact on the effectiveness of learnings and the extent to which they are sustained and translated into clinical practice. This study, which investigated the impact of a first year Aboriginal health unit on midwifery students’ knowledge and attitudes, found significant pre- and post-unit differences, but also observed a steady decline in the impact of the content in subsequent years. While a number of factors might be implicated, the pattern of findings suggests that if an Aboriginal health unit is located in the first year of a program, vertical integration is necessary to consolidate knowledge acquired and the positive shift in attitudes observed. Suggestions for additional midwifery-specific Aboriginal content and strong student interest in more interaction with Aboriginal women in clinical and community settings provide opportunities for future curriculum development. If harnessed, this interest would enhance students’ preparation to deliver culturally secure care to Aboriginal women and maximise the early gains observed in this study.

## Endnotes

^**a**^In the Australian context the term ‘Indigenous’ refers to Aboriginal and Torres Strait Islanders and appears in this paper when it is included in the title of reports or referred to in citations. The term ‘Aboriginal and Torres Strait Islander’ is frequently abbreviated to ‘Aboriginal’ and that is the usage in this paper, except where the full term is used in reports or citations. These terms are often used interchangeably by government bodies, the academy and by Aboriginal people themselves and hence it is not possible to impose consistency.

^b^One of the authors (RDT) developed and taught the original unit, together with Aboriginal colleagues.

^c^Grandmothers’ law relates to intergenerational knowledge and skills concerning women’s business including birthing, pregnancy and the care of children [31].

## References

[1] Universities Australia (2011). Guiding principles for developing Indigenous cultural competency in Australian Universities.

[2] Saggers S, Walter M, Gray D, Thackrah R, Scott K (2011). Culture, history and health. Indigenous Australian health and cultures: an introduction for health professionals.

[3] Betancourt J, Green A (2010). Linking cultural competence training to improved health outcomes: perspectives from the field. Acad Med.

[4] Ewen S, Paul D, Bloom G (2012). Do Indigenous health curricula in health science education reduce disparities in health care outcomes?. Med J Aust.

[5] Lie D, Lee-Rey E, Gomez A, Bereknyei S, Braddock C (2010). Does cultural competency training of health professionals improve patient outcomes? A systematic review and proposed algorithm for future research. J Gen Intern Med.

[6] Phillips G (2004). CDAMS Indigenous health curriculum framework.

[7] Medical Deans Australia and New Zealand Inc and the Australian Indigenous Doctors’ Association Ltd. National medical education review. Canberra: The Australian Indigenous Doctors’ Association; 2012.

[8] Indigenous Nursing Education Working Group (2002). ‘gettin em n keepin em’.

[9] Health Workforce Australia. www.hwa.gov.au/resources/publications.

[10] Paul D, Carr S, Milroy H (2006). Making a difference: the early impact of an Aboriginal health undergraduate medical curriculum. Med J Aust.

[11] Crampton P, Dowell A, Parkin C, Thompson C (2003). Combatting effects of racism through a cultural immersion medical education program. Acad Med.

[12] Paul D, Allen C, Edgill P (2011). Turning the corner. Assessment: a key strategy to engagement and understanding in Indigenous health. Focus Health Prof Educ.

[13] Rasmussen L (2001). Towards reconciliation in Aboriginal health: initiatives for teaching medical students about Aboriginal issues.

[14] Downing R, Kowal E (2010). Putting Indigenous cultural training into nursing practice. Contemp Nurse.

[15] Flavell H, Thackrah R, Hoffman J (2013). Developing Indigenous cultural competence: a model for implementing Indigenous content into curricula. J Teach Learn Grad Empl.

[16] McDermott D, Sjoberg D (2012). Managing a diverse student discomfort with an Indigenous health curriculum. In: Leaders in Indigenous Medical Education Network, editor. LIME good practice case studies.

[17] Nash R, Meiklejohn B, Sacre S (2006). The Yapunyah project: embedding Aboriginal and Torres Strait Islander perspectives in the nursing curriculum. Contemp Nurse.

[18] Thackrah R, Thompson S (2013). Confronting uncomfortable truths: receptivity and resistance to Aboriginal content in midwifery education. Contemp Nurse.

[19] Department of Families, Health, Community Services and Indigenous Affairs (2013). Closing the Gap Prime Minister’s Report.

[20] Kildea S, Van Wagner V (2012). ‘Birthing on Country’ maternity service delivery models: a rapid review.

[21] Pedersen A, Barlow F (2008). Theory to social action: a university-based strategy targeting prejudice against Aboriginal Australians. Aust Psychol.

[22] Liamputtong P (2011). Qualitative Research Methods.

[23] Australian Nursing and Midwifery Council (2006). National Competency Standards for the Midwife.

[24] Te Kaunihera Tapuhi o Aotearoa, Council of New Zealand (2011). Guidelines for Cultural Safety, the Treaty of Waitangi, and Maori Health in Nursing Education and Practice.

[25] Belfrage M (2007). Why “culturally safe” health care?. Med J Aust.

[26] Health Department of Western Australia (2003). Aboriginal Cultural Security: A Background paper.

[27] Houston S, Keleher H, MacDougall C (2009). Cultural security as a determinant of Aboriginal health. Understanding Health.

[28] McDermott D, Gabb D (2010). Exploring the Unspoken: Racism, Indigenous Australians and Psychologist Professional Development.

[29] McDermott D (2012). Can we educate out of racism?. Med J Aust.

[30] Paul D, Leaders in Indigenous Medical Education Network (2012). All or nothing? The value of a complete package - a horizontally and vertically integrated Aboriginal health curriculum. LIME good practice case studies.

[31] Kildea S, Wardaguga M, Selin H, Stone P (2009). Childbirth in Australia: Aboriginal and Torres Strait Islander Women. Childbirth across cultures: ideas and practices of pregnancy, childbirth and the postpartum.

